# Detection of IL-36γ through noninvasive tape stripping reliably discriminates psoriasis from atopic eczema

**DOI:** 10.1016/j.jaci.2018.04.031

**Published:** 2018-09

**Authors:** Anna Berekméri, Anne Latzko, Adewonuola Alase, Tom Macleod, Joseph S. Ainscough, Philip Laws, Mark Goodfield, Andrew Wright, Philip Helliwell, Sara Edward, Gordon D. Brown, Delyth M. Reid, Joerg Wenzel, Martin Stacey, Miriam Wittmann

**Affiliations:** a Leeds Institute of Rheumatic and Musculoskeletal Medicine (LIRMM), University of Leeds, Leeds, United Kingdom; c Faculty of Biological Sciences, School of Molecular and Cellular Biology, University of Leeds, Leeds, United Kingdom; b National Institute of Health Research (NIHR), Leeds Biomedical Research Centre (BRC), Leeds Teaching Hospitals, Leeds, United Kingdom; d Bradford Teaching Hospital Foundation Trust, Bradford, United Kingdom; e Centre for Skin Sciences, University of Bradford, Bradford, United Kingdom; f St James's Institute of Oncology, St James's University Hospital, Leeds, Leeds, United Kingdom; g Medical Research Council Centre for Medical Mycology at the University of Aberdeen, Aberdeen Fungal Group, Institute of Medical Sciences, Aberdeen, United Kingdom; h Department of Dermatology and Allergy, University Hospital Bonn, Bonn, Germany

To the Editor:

Inflammatory skin reactions, regardless of their distinct underlying pathophysiologic mechanisms, can often present with similar morphology. Although typical eczema and lesions of plaque psoriasis are easily distinguishable by experienced dermatologists, these 2 diseases often cause diagnostic difficulties when inflammation is minimal or located in certain anatomical regions, including flexures, the scalp, auricular or palmoplantar areas. In these cases diagnosis can be problematic, in particular in the primary care setting, where misdiagnosis can lead to delayed appropriate treatment, overuse of antibiotics, or both.

Histopathology, which is the current gold standard in diagnosing challenging cases, is invasive, costly, and often unavailable in the primary care setting. Furthermore, histopathologic differentiation of psoriasis from eczema in some anatomical localizations, such as the palmoplantar region, is difficult.^1^ Unfortunately, there are also no reliable blood-derived diagnostic biomarkers that cover the wide range of clinical phenotypes and disease severities, and although RNA signatures from lesional skin biopsy specimens have been described for atopic dermatitis (AD) and psoriasis, mRNA analysis can be costly and labor intensive. Therefore a simple, noninvasive, and reliable diagnostic approach would be of great clinical benefit. To address this need, this study uses a noninvasive, tape-stripping, and ELISA-based approach to investigate potential protein biomarkers that are able to discriminate eczematous from psoriatic inflammation presenting with a range of severities.

Because the epidermis is a significant source of chemokines, inflammatory lesions from patients with psoriasis and those with AD were sampled initially by means of tape stripping and analyzed for the neutrophil-recruiting chemokines CXCL1 and IL-8, as well as CCL20, which recruits IL-17/IL-22–producing cells (see the Methods section in this article's Repository at www.jacionline.org for detailed methodology).^2^ Although these chemokines were found at significantly greater levels in tape-stripping samples from lesions of patients with psoriasis compared with those with AD, receiver operating characteristic (ROC) curve analysis indicated that they would not be ideal as strong discriminators of the 2 conditions (IL-8: area under the curve [AUC], 0.83; SE, 0.0523; 95% CI, 0.726-0.931; CXCL1: AUC, 0.796; SE, 0.049; 95% CI, 0.7-0.891 [Fig 1, *B*]; CCL20: AUC, 0.82; SE, 0.045; 95% CI, 0.731-0.905). For all 3 chemokines, no or very low levels of protein were detected in healthy and nonlesional samples.

**Fig 1:**
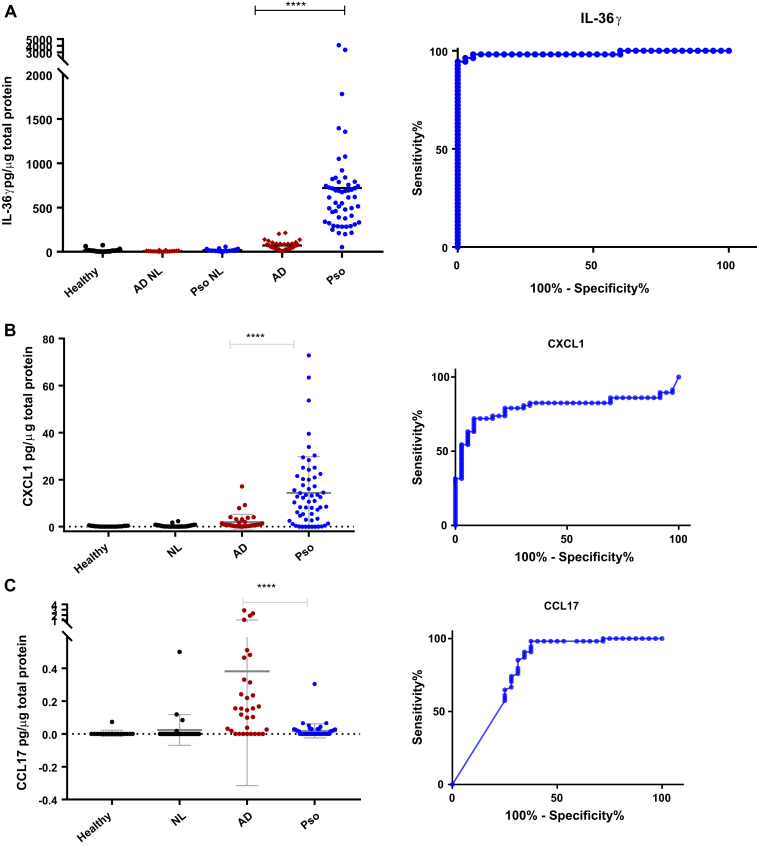
Comparison of IL-36γ **(A)**, CXCL1 **(B)**, and CCL17 **(C)** levels in tape samples from psoriatic and atopic eczema lesions. Tape stripping was performed on healthy volunteers and both lesional and nonlesional *(NL)* skin from patients with clinically diagnosed AD or psoriasis *(Pso)*. Tape-derived cytokine levels were normalized to total protein content. ROC curves are depicted for all 3 parameters comparing samples from patients with eczema and those with psoriasis. ****P < .0001.

Because high levels of IL-36γ mRNA and protein have been reported in lesions of patients with psoriasis,^3^, ^4^ IL-36γ from tape samples was quantitated through use of a novel in-house sandwich ELISA (see the Methods section in this article's Online Repository). IL-36γ showed a trend for increased levels in patients with AD compared with levels seen in nonlesional or healthy skin (mean, 71/13.5/57.5 pg/μg total protein, respectively). However, IL-36γ levels within psoriatic lesions were significantly greater than those in AD lesions (mean, 719 vs 71 pg/μg total protein; Fig 1, *A*). No difference in IL-36γ expression was observed in healthy versus nonlesional psoriatic skin taken from ventral forearm areas.

A ROC curve (AUC, 0.987; SE, 0.0114; 95% CI, 0.965-1.01) was plotted to determine the sensitivity and specificity of IL-36γ in a diagnostic approach (Fig 1, *A*). At an optimal cutoff level of 214 pg/μg total protein (Youden index, 0.944), IL-36γ specificity was calculated as 100% (95% CI, 90% to 100%), and sensitivity was calculated as 94.44% (95% CI, 84.61% to 98.84%). This suggests that IL-36γ has an excellent potential as a diagnostic marker for psoriatic inflammation. When compared with CXCL1, the increased expression of IL-36γ is far more consistent, being found across all patients with psoriasis. We also measured IL-36γ expression in a range of other skin pathologies, including fungal infection, lichen planus, systemic-, subacute cutaneous-, and chronic discoid lupus erythematosus, all of which showed expression levels less than the cutoff level of 214 pg/μg total protein (data not shown).

Both CCL27 and CCL17 have been suggested as potential biomarkers for AD.^5^ CCL17 preferentially attracts IL-4/IL-13–producing lymphocytes, whereas CCL27 is involved in the homing of memory T cells to the skin.^6^ Epidermal tape stripping demonstrated CCL17, but not CCL27, to be of value in identifying AD (Fig 1, *C*). CCL27 levels were increased in both psoriatic and AD lesions. In those cases in which CCL17 was detectable, it pointed to an underlying AD inflammation (AUC, 0.79; SE, 0.058; 95% CI, 0.676-0.903). However, unlike IL-36γ, which was detectable in all psoriatic lesions tested, a significant number of AD samples (25%), as well as 57.4% of psoriatic samples, did not show any measurable CCL17.

The patient cohort (see Table E1 in this article's Online Repository at www.jacionline.org) included in our analysis showed prototypic plaque psoriasis or AD. However, to further illustrate the potential of tape-collected IL-36γ as a diagnostic approach for psoriasis, we investigated clinical cases of unclear diagnosis. In each case the diagnostic value of IL-36γ was correct when including dermatohistopathologic results. As an example, we show 2 clinical cases (for details, see the Methods section in this article's Online Repository).

The first case was given a clinical diagnosis of and treated for palmoplantar eczema in our dermatology department. However, analysis of tape stripping showed IL-36γ levels of 960 pg/μg total protein, which are indicative of psoriasis. This fact and subsequent dermatohistopathologic analysis and changes in clinical features were supportive of a corrected diagnosis to that of psoriasis (Fig 2, *A*).

**Fig 2:**
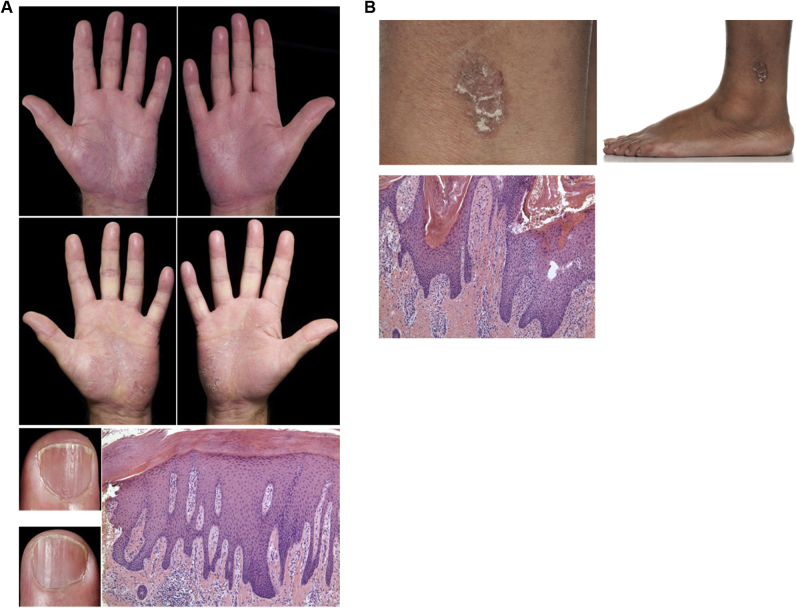
Tape stripping for the diagnosis of difficult clinical cases. Shown are pictures of 2 clinical presentations in which the clinical diagnosis was unclear or challenging. Based on an IL-36γ cutoff level of 214 pg/μg total protein, tape stripping results indicated psoriatic inflammation for case A but not case B. Histopathologic examination confirmed the results. **A,** Palmar psoriasis was misdiagnosed initially as hand eczema. **B,** Chronic eczema. For further details on Fig 2, see this article's Online Repository at www.jacionline.org.

The other case was a patient with symptoms of joint pain in addition to erythrosquamous skin lesions. For the referring rheumatologist, a confirmation of these skin lesions as psoriasis would guide future diagnostic and treatment pathways in the direction of psoriatic arthritis. However, tape stripping did not confirm psoriatic inflammation, and the diagnosis for the skin lesions was confirmed to be chronic eczema (Fig 2, *B*).

Numerous soluble mediators, cell-surface molecules, and intracellular proteins have been described previously to be upregulated in psoriatic lesions.^2^ In our noninvasive approach we could confirm that levels of the neutrophil chemoattractants IL-8 and CXCL1,^7^ as well as CCL20, the chemoattractant for IL-17–producing cells, are increased in psoriatic compared with eczema lesions. Contrary to work showing reduced expression of CCL27 in patients with psoriasis^8^ and the value of CCL27 as a biomarker for AD,^9^ we did not find significant differences in the amount of this chemokine. The differences between our findings and those of some others is likely to be due to the method of sampling.

In conclusion, although the effects of other factors, such as systemic and topical therapeutics, age, sun exposure, and lesion chronicity, need to be investigated in future studies, the results presented here confirm^4^ IL-36γ to be a robust, specific, and reliable biomarker for psoriatic inflammation that outperforms previously reported biomarkers and is likely to withstand all challenges in real-life primary and secondary dermatologic care.

## Figures and Tables

**Table E1:** Demographic characteristics of patients

	Patients with AD	Patients with psoriasis	Healthy subjects
Sex			
Male	28%	51%	38%
Female	72%	49%	62%
Age (y)			
Mean	42	47	37
Range	21-69	18-67	25-50

**Table E2:** Comparison of local severity scores of patients with AD and psoriasis

	Patients with AD	Patients with psoriasis
Mean erythema score	2.40	2.61
Mean infiltration score	2.37	2.14
Mean scaling score	1.88	2.18
Mean total lesion score	2.23	2.31

A local severity score was determined by experienced dermatologists for all patients with eczema and psoriasis included in the study.

## Methods

### Patients

Patients with a clinical diagnosis of plaque psoriasis or AD presenting with “typical” morphology, as diagnosed by consultant dermatologists, were included in the study. Hanifin and Rajka diagnostic criteria were followed for eczema, and diagnosis of plaque psoriasis was based on traditional pattern recognition combined with patient history. Patients' demographics are shown in Table E1.

This study was approved by NRES Committee North East-York (ILTIPP study: REC 14/NE/1199, ALPHA study: REC 14/YH/1259) and for recruitment of volunteers by the ethics committee of the University of Leeds (BIOSCI 14-001). All participants provided written informed consent before participation.

Overall disease activity differed significantly and was not collected for analysis because the focus was on the individual lesion. A local lesion severity score was assessed based on Psoriasis Activity and Severity Index items (covering the range of 0-4) by specialist dermatologists before sample collection (see Table E2). Patients receiving systemic immunomodulatory therapy or biologics were excluded apart from 1 patient receiving anti–TNF-α therapy (etanercept), which had lost efficacy, and 1 patient receiving low-dose acitretin therapy. Where possible, preference was given to patients who have not used topical corticosteroids in the sampled area for at least 48 hours. In the eczema group patients were considered atopic if presenting with clinical symptoms of immediate-type reactions to allergens and/or the presence of specific IgE, as measured by a routine RAST in serum samples.

### Samples

Tape stripping was performed on lesional and nonlesional skin (ventral forearm) at routine dermatology and combined dermatology/rheumatology clinics and processed by using an improved tape-stripping protocol based on previously published methodology.E1, E2 D-Squame adhesive discs of 3.8 cm^2^ (CuDerm, Dallas, Tex) were used. Choice of sample site was guided by location of lesions, but preference was given to the hands, arms, and upper trunk area. Only nonerosive nonoozing lesions were tape stripped. The first tape was discarded, and 10 subsequent tapes from the same location were collected and immediately stored on dry ice for transportation or stored at −80°C until protein was extracted.

### Protein extraction and concentration determination

Frozen tapes were transferred into 1.5 mL of lysis buffer (20 mmol/L Tris [pH 7.4], 150 mmol/L NaCl, 1% Triton X-100, 5 mmol/L EDTA, and 1× protease inhibitor cocktail; Roche, Welwyn Garden City, United Kingdom). Tubes containing tapes were left on ice for 30 minutes before sonication cycles of 3 times 20 seconds with a 20-second interval on ice between each sonication. The extracts were centrifuged at 15,000*g* for 10 minutes, and the protein concentration of each sample was determined by using the bicinchoninic acid assay (Life Technologies, Paisley, United Kingdom).

### Generation of human IL-36γ ELISA and measurement of soluble mediators

To generate mAbs against human IL-36γ, C57B/6 mice or Sprague Dawley rats were immunized twice with recombinant IL-36γ Ser_18_-Asp_169_ first in complete Freund adjuvant and subsequently in incomplete Freund adjuvant. Aqueous boosts 3 to 4 days before fusions were administered intraperitoneally with the same protein in PBS. Spleen cells were fused with the myeloma cell lines Y3-AG 1.2.3 (rat) or SP2/O-Ag14 (mouse), as appropriate, by using a method similar to the original described by Kohler and Milstein.^E3^ Hybridoma supernatants were screened against IL-36γ– or appropriate control–coated immunosorbent plates, and selected fusion wells were cloned twice to ensure monoclonality.

Characterization of purified mAbs indicated that the antibody generated by mouse hybridoma B5A2 performed effectively as a capture antibody, and the antibody generated by rat hybridoma HCL17 performed effectively as a detection antibody by using sandwich ELISA. Antibodies were subsequently purified with either protein A or protein G affinity chromatography. The antibody HCL17 was then biotinylated with EZ link NHS-LC-biotin (Thermo Scientific, Waltham, Mass), according to the manufacturer's instructions.

### ELISA

Immunosorbent 96-well ELISA plates (Nunc Life Technologies, Paisley, United Kingdom) were coated with 2 μg/mL B5A2 capture antibody in PBS at 4°C overnight. Plates were then washed 3 times with 0.1% Tween 20/PBS and blocked for 1 hour in 2% BSA in 0.1% Tween-20/PBS. Samples were incubated subsequently for 1 hour at room temperature before washing and incubation with 1 μg/mL HCL17 biotinylated detection antibody for 1 hour. Plates were then washed and incubated with streptavidin–horseradish peroxidase (BioLegend, London, United Kingdom) for 20 minutes. After washing, TMB was used as a chromogenic substrate (Thermo Scientific). The reaction was stopped with 2N H_2_SO_4_, and OD was measured at 450 nm. A standard curve was obtained from a 7-point serial dilution of protein standard and used to calculate IL-36γ concentrations. CCL27 from the tape-stripping samples was quantified by means of ELISA (Bio-Techne, Abingdon, United Kingdom), according to the manufacturer's protocol.

### Multiplex bead-based quantification assays

Cytokines (CCL20, CXCL1, and IL-8) from tape-stripping samples were measured by using bead-based immunoassay purchased from BioLegend. The assay was carried out according to the manufacturer's instructions, and fluorescent beads were acquired by using flow cytometry (LSRII; BD Biosciences). The results were analyzed with LEGENDplex analysis software (BioLegend).

### Histopathology

Routine diagnostic punch biopsy specimens were taken from lesional skin, fixed in 10% formaldehyde, embedded in paraffin, stained with hematoxylin and eosin, and evaluated by experienced dermatopathologists.

### Statistical analysis

Results were analyzed with GraphPad Prism software, version 7.00 (GraphPad Software, La Jolla, Calif). Results for each group are depicted as means ± SDs. Data were analyzed by using 1-way ANOVA followed by the Tukey multiple comparison test to determine statistically significant differences between groups. Sensitivity and specificity analysis was performed by using ROC curve analysis. The optimal cutoff level was determined according to the Youden index.

### Clinical cases

Case A, a 61-year-old male patient, received a diagnosis of chronic palmoplantar eczema based on clinical appearance and symptoms (Fig 2, *A*). There were no signs of eczema or psoriasis elsewhere on the body. The patient had no personal history of atopy; family history was positive for allergic asthma and negative for psoriasis. Patch testing revealed a positive reaction to methylchloroisothiazolinone, exposure to which the patient avoided subsequently. The patient's total IgE level was 767 kU/L without detectable specific IgE to inhaled allergen mix. Mycology microscopy and culture results were negative for fungal infection. The patient showed very little to no benefit from immersion PUVA therapy and presented little response to 30 mg/d oral alitretinoin. During the disease course, lesions gained a slightly different appearance as coarser scales developed. Tape-stripping samples were taken before a diagnostic biopsy was obtained from a right palmar lesion. Results were highly suggestive of the diagnosis of psoriasis, with significantly enhanced levels of IL-36γ (IL-36γ, 959.3 pg/μg; CXCL1, 15.9 pg/μg; and IL-8, 29.2 pg/μg; CCL17 was not detected). Histopathologic examination from the same site confirmed the diagnosis of psoriasis. Subsequently, re-examination of the patient revealed some nail pitting (Fig 2, *A*), a symptom associated with psoriatic inflammation.

Case B, who was referred by the rheumatology department, presented with unclassified polyarthralgia and some skin symptoms. The presence of relatively well circumscribed margins and a thick adherent scale included psoriasis as a differential diagnosis (Fig 2, *B*). However, low expression of IL-36γ in tape-stripping samples did not suggest psoriasis (IL-36γ, 25.2 pg/μg; CXCL1, 0.12 pg/μg; and IL-8, 0.25 pg/μg; CCL17 levels were less than detection levels). Subsequent histopathologic examination confirmed the diagnosis of eczema.

### Detailed legend of Fig 2

**A,** *Upper pictures*, Typical clinical signs of eczema: palmar erythema with moderate infiltration of the skin, deeply seated vesicles with discrete erosions, and fine scaling associated with intense itchiness. *Lower pictures*, The same patient 3 months later presented a change in clinical phenotype with the presence of rare intact pustules accompanied by accentuated scaling and typical nail changes showing pitting and ridging. Histopathologic examination shows regular epidermal hyperplasia, with clubbing and anastomosis of the elongated rete ridges associated with suprapapillary plate thinning. Mild, patchy, perivascular infiltrate composed of lymphocytes without eosinophils and mild exocytosis is present. The granular layer is still present as well. However, there is some parakeratosis in which collections of neutrophils are identified (hematoxylin and eosin stain; high-power magnification ×400). **B,** *Upper pictures*, Relatively well-demarcated, slightly erythematous, intensely itchy lesion covered by thick adherent scales in the left lateral supramalleolar localization of a 51-year-old female patient. Histopathologic examination showed irregular epidermal hyperplasia in association with hyperkeratosis and hypergranulosis. Patchy epidermal spongiosis with focal lymphocytic exocytosis can be seen. There is no club-shaped elongation of the rete ridges, and neutrophils are not identified. The features are in favor of those of a lichenified eczema (hematoxylin and eosin stain; high-power magnification ×400).
